# Probiotic potential of *Phocaeicola coprocola* in modulating learning and memory behaviors in the honeybee model

**DOI:** 10.3389/fmicb.2025.1479992

**Published:** 2025-06-20

**Authors:** Mengqi Xu, Xiaohan Zhang, Xi Luo, Guanzhou Zhou, Nana Zhang, Xiaoyan Chi, Rongrong Ren, Lihua Peng, Gang Sun, Yunsheng Yang

**Affiliations:** ^1^Microbiota Laboratory, Clinical Division of Microbiota, Department of Gastroenterology and Hepatology, The First Medical Center, Chinese PLA General Hospital, Beijing, China; ^2^Medical School of Chinese PLA, Beijing, China; ^3^Medical School of Nankai University, Tianjin, China; ^4^Department of Pediatrics, The First Medical Center, Chinese PLA General Hospital, Beijing, China; ^5^National Clinical Research Center for Geriatric Diseases, Chinese PLA General Hospital, Beijing, China

**Keywords:** *Phocaeicola coprocola*, *Apis mellifera*, cognitive performance, microbiota-gut-brain axis, glycerophospholipid metabolism

## Abstract

**Introduction:**

Gut microbial therapy has emerged as a prominent research topic for brain function and disorders. The depletion of *Phocaeicola coprocola* has been reported in various brain-related conditions, suggesting its possible neuroprotective and cognitive benefits. However, its functional roles and underlying mechanisms remain poorly understood.

**Methods:**

We evaluated the effects of *P. coprocola* on cognitive performance using the honeybee (*Apis mellifera*) as a novel model for the microbiota–gut–brain axis. Honeybees with a standardized gut microbiota served as the control group, while those supplemented with *P. coprocola* comprised the treatment group. Olfactory learning and memory were assessed using classical conditioning assays. Gut microbial composition was analyzed using full-length 16S rRNA gene sequencing based on PacBio SMRT technology, and metabolic profiling was conducted using untargeted LC–MS/MS analysis.

**Results:**

*P. coprocola* supplementation significantly improved cognitive performance, with learning success rates of 74.13% in the treatment group versus 50.85% in controls (*p* = 0.0093). This intervention also led to increased gut diversity (Shannon index, *p* = 0.0079). Metabolomic analysis revealed substantial alterations in intestinal lipid metabolism, particularly in glycerophospholipid pathways (*p* = 0.0002). Furthermore, the increase in protective lipid molecules, such as phosphatidylcholine, glycerophosphocholine, and glycerophosphoethanolamine, was strongly correlated with *Gilliamella apicola*, *Bifidobacterium asteroides*, and *Bombella apis*.

**Discussion:**

*P. coprocola* has potential as a probiotic candidate for modulating cognition-related processes via gut microbial and metabolic interactions. Moreover, the honeybee model offers a valuable platform for preclinical investigation of microbiota-gut-brain relationships and probiotic screenin.

## 1 Introduction

The extensive genetic and metabolic potential of gut microbiota underscores its crucial role in human health and disease. Dysbiosis of the microbiota has been linked to neuropsychiatric and neurological disorders, and the concept of the microbiota-gut-brain axis is increasingly being recognized (Wang et al., 2023; Loh et al., 2024). Microorganisms can function in bidirectional communication between the gut and brain via the immune system, neuroendocrine system, neurotransmitters and metabolites, vagus nerve, and other pathways (Chakrabarti et al., 2022). Targeting the modulation of gut microbiota has emerged as an effective therapeutic strategy for neuropsychiatric and neurological disorders. Our previous study explored the efficacy and safety of fecal microbiota transplantation (FMT) in patients with Tourette syndrome. We observed a significant reduction of *Phocaeicola coprocola* (formerly *Bacteroides coprocola*) in these patients compared to healthy controls. Longitudinal analysis indicated that restoring *P. coprocola* levels through FMT correlated with improvements in tic symptoms (Zhao et al., 2020). *P. coprocola* is a Gram-negative, rod-shaped symbiotic anaerobe that commonly inhabits the lower digestive tract. Depletion of *P. coprocola* has also been observed in other brain disorders, such as attention-deficit/hyperactivity disorder (Wang et al., 2020), Parkinson’s disease (Petrov et al., 2017), and multiple system atrophy (Wan et al., 2019). *In vitro* evidence has shown that the intestinal epithelial barrier protects the anti-inflammatory cytokine-releasing properties of *P. coprocola* (Cuffaro et al., 2021). As mentioned above, we hypothesized that *P. coprocola* could improve brain function via the gut-brain pathway.

Over the past 5 years, honeybees have emerged as a potential model for research on the microbiota-gut-brain axis (Zheng et al., 2018; Li et al., 2021; Zhang et al., 2022; Chang et al., 2022; Li et al., 2023; Zeng et al., 2024). The honeybee microbiota is simple and specific and is dominated by five core bacterial clades: *Lactobacillus* Firm-5, *Lactobacillus* Firm-4, *Bifidobacterium* species, *Snodgrassella alvi*, and *Gilliamella apicola* (Zheng et al., 2018; Alberoni et al., 2021; Zheng et al., 2020). Honeybees exhibit intricate social behaviors and cognitive functions with well-established behavioral assessment procedures, such as learning and memory assays based on the proboscis extension reflex (PER) (Matsumoto et al., 2012). Furthermore, the cost-effectiveness and short experimental duration of honeybees make them economically viable and practical preclinical models.

The depletion of a common symbiotic bacterium, *P. coprcola*, implies that it may have a probiotic role in neuropsychiatric and neurological disorders (Zhao et al., 2020; Wang et al., 2020; Petrov et al., 2017; Wan et al., 2019); however, there is a lack of relevant research. This study introduced honeybees as a complementary preclinical platform to investigate the potential neuromodulatory capacity of *P. coprocola*, thereby providing preclinical proof-of-concept for its prioritization in probiotic development pipelines.

## 2 Materials and methods

### 2.1 Ethics statement

Although there are no formal guidelines for the care and use of insects in research, all experimental procedures involving honeybees were conducted in accordance with best practices to minimize suffering and distress. Bees were provided with adequate daily care, including proper nutrition, throughout the study. Tissue collection was performed humanely by gently capturing the bees and euthanizing them immediately using CO₂ anesthesia prior to dissection. This study did not involve endangered or protected species, and all efforts were made to ensure ethical treatment in compliance with institutional standards.

### 2.2 Generation of honeybees with standardized gut microbiota

The honeybee workers (*Apis mellifera*) used in this study were obtained from Beijing Jinhai Lake Happy Apiary. All the bees used in this study were from the same colony. Germ-free (GF) bees were obtained as previously described by our team (Li et al., 2023) and Zheng et al. (2019), with some modifications. Late-stage pupae were manually removed from brood frames and placed in sterile plastic bins for further maturation at 35°C and 50% humidity until eclosion. Honeybee gut samples were homogenized and cultured on brain heart infusion agar to confirm the absence of bacterial growth. Groups of 20–25 newly emerged GF bees (day 0) were placed into a cup cage and fed a mixture of 5 μL of gut homogenate stock from 200 wild bees, 1 mL of 1 × phosphate-buffered saline (PBS), 1 mL of sterilized sucrose solution (50%, w/v), and 0.3 g of sterilized pollen for 48 h to standardize the gut microbiota of bees (day 2). Bees from the same cup cage were considered one replicate for each group. Each group had three replicate cup cages, containing 20–25 bees per cup.

### 2.3 Bacterial treatments

*Phocaeicola coprocola* (DSM 17136) was used in this study. The full-length 16S rRNA gene sequence of strain DSM 17136 used in this study is provided in the Supplementary materials. *P. coprocola* was cultivated in brain heart infusion supplemented with 0.05% L-cysteine (HCl), 5 mg/L hemin, and 0.01% vitamin K1 under anaerobic conditions for 48 h. On the day of treatment, *P. coprocola* was washed twice and resuspended in an equal mixture of 1 × PBS and sterilized sucrose solution (50%, w/v) at a final OD600nm of 0.5.

**Figure 1:**
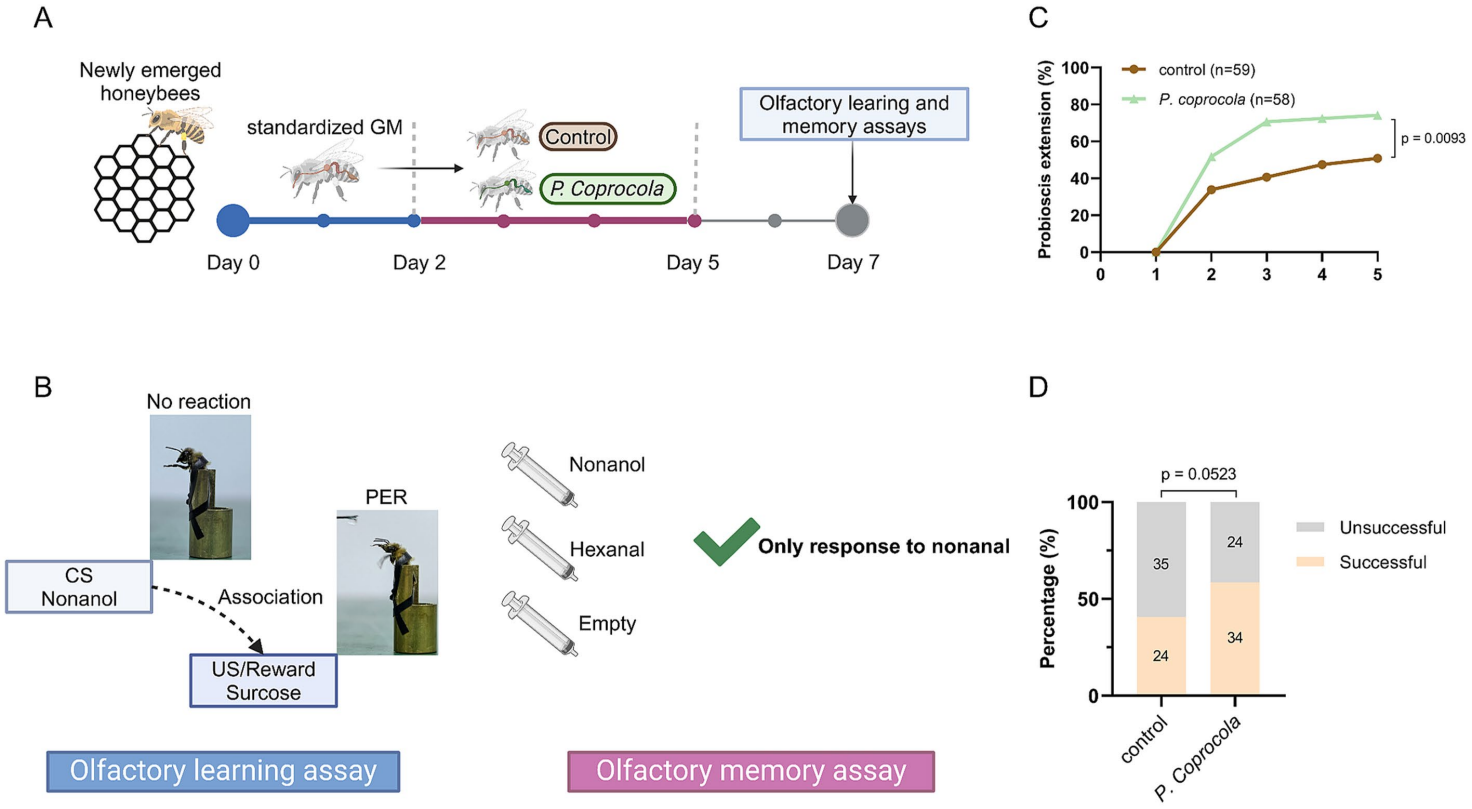
*Phocaeicola coprocola* supplementation improves learning and memory performance in honeybees. **(A)** Honeybees were subjected to a standardized gut microbiota, followed by a 3-day exposure to *P. coprocola* or blank control and an additional 2-day washout period. The learning and memory assays were conducted on day 7. **(B)** Flow diagram of olfactory learning and memory assays. When presented with sucrose solution, an unconditional stimulus (US), PER, occurred in honeybees. Honeybees were mounted on a modified 0.8 mm wide bullet shell with sticky tape restraining harnesses. Nonanol odor, the conditional stimulus (CS), was given before the US and reward. We assessed whether honeybees could associate the nonanol odor with sucrose rewards. Olfactory learning memory assays were conducted 2 h after training. Two odor stimuli (nonanol or hexanal) were randomly administered, with a clean syringe presented subsequent to each administration to eliminate visual stimulus effects. Bees that extended the proboscis only to a nonanol odor were considered successful. **(C)** Learning curves for positively rewarded CS. **(D)** Memory test performance 2 h after training. Sample sizes are indicated in bars. Learning and memory data were analyzed using two-sided Chi-square tests (exact *p*-values shown in the panels). Figure was created using BioRender.com.

Honeybees with standardized gut microbiota were divided into two groups: (1) control and (2) *P. coprocola*-colonized (*P. coprocola* group). In the control group, the bees were fed sterilized pollen and an equal mixture of 1 × PBS and sterilized sucrose solution (50%, w/v) until day 7. For the *P. coprocola* group, the bees were fed sterilized pollen and a bacterial suspension as described above for 72 h (the bacterial suspension was renewed every 12 h), followed by an equal mixture of 1 × PBS and sterilized sucrose solution (50%, w/v) until day 7. The overall design of the supplementation and behavioral assay schedule is illustrated in Figure 1A.

### 2.4 Olfactory learning and memory assays

Olfactory learning and memory assays were conducted on day 7 as previously described (Chang et al., 2022; Li et al., 2023), with some modifications. Nonanol (training odor; Sigma-Aldrich, St. Louis, MO, United States) and hexanal (negative control; Macklin, Shanghai, China) were used as odor sources.

The bees were starved for 2 h before the test by removing the sugar syrup and pollen from the cup cage. They were then mounted on a modified 0.8 mm wide bullet shell with sticky tape restraining harnesses. The experiment was conducted using a stable light source at room temperature. Each bee was checked for an intact PER by touching the antennae with a 50% sucrose solution without subsequent feeding. Bees that did not show a PER for sucrose were removed from further experiments. In the olfactory learning test, bees were trained for five rounds at 10-min intervals to associate the odor of nonanol as the conditioned stimulus with a 50% sucrose solution reward as the unconditioned stimulus. Briefly, the harnessed bee was introduced into the arena for 6 s to acclimate to the experimental setting and was positioned in front of an exhaust fan to avoid odor accumulation in future trials. Subsequently, the nonanol odor was presented before the antennae for 4 s. A 0.4 μL droplet of sucrose solution was then administered to the bee via a syringe needle for 3 s, making direct contact with the proboscis to elicit PER. There was a one-second overlap between the nonanol odor and the sucrose solution. From the second training round, bees that recognized the nonanol odor and extended the proboscis before giving sucrose were considered successful in learning assays. After five training rounds, bees that did not respond to the nonanol odor were considered unsuccessful. The bees were kept unfed in the dark for 2 h and then tested for olfactory memory. Two odor stimuli (nonanol or hexanal) were randomly administered, with a clean syringe presented subsequent to each administration to eliminate visual stimulus effects. Bees that extended the proboscis only to a nonanol odor were considered successful. The olfactory conditioning protocol is illustrated in Figure 1B.

### 2.5 Tissue collection

Honeybee stingers were carefully removed and the whole gut was dissected using sterilized tweezers. The dissected guts were collected into 1.5 mL centrifuge tubes and stored at −80°C until 16S rRNA gene sequencing or metabolomic analysis.

### 2.6 Gut microbiota DNA extraction and 16S rRNA gene sequencing

Genomic DNA of the microbial community was extracted from the dissected gut samples using the FastPure Stool DNA Isolation Kit (MJYH, Shanghai, China), according to the manufacturer’s instructions. The DNA extract was analyzed on a 1% agarose gel, and DNA concentration and purity were determined using a NanoDrop 2000 UV–vis spectrophotometer (Thermo Scientific, Wilmington, NC, United States). The 16S rRNA genes were amplified using the universal bacterial primers 27F (5′-AGRGTTYGATYMTGGCTCAG-3′) and 1492R (5′ -RGYTACCTTGTTACGACTT-3′). The polymerase chain reaction products were purified using AMPure® PB beads (Pacifc Biosciences, CA, United States) and quantified using a Synergy HTX (Biotek, USA). Purified products were pooled in equimolar amounts, and the DNA library was constructed using the SMRTbell Prep Kit 3.0 (Pacifc Biosciences, CA, United States) according to the manufacturer’s instructions. Purified SMRTbell libraries were sequenced using a PacBio Sequel IIe System (Pacific Biosciences, CA, United States) by Majorbio Bio-Pharm Technology Co. Ltd. (Shanghai, China).

PacBio raw reads were first processed using SMRTLink software (version 11.0) to generate high-quality HiFi reads. Circular Consensus Sequences (CCS) were obtained using the ccs module, with filtering criteria set to a minimum of three full passes (minFullPass = 3) and ≥99% predicted accuracy (minPredictedAccuracy = 0.99). Full passes refer to the number of complete circular traversals of the insert sequence; only reads with ≥3 complete passes were retained. Adapter-trimmed and barcode-demultiplexed CCS reads were further filtered by length, retaining only sequences between 1,000–1,800 bp. Sequences were corrected for orientation using 5′ and 3′ primer information and then had primer sequences removed. Clean reads were saved in FASTQ format. Amplicon sequence variants (ASVs) were inferred using the DADA2 plugin in QIIME2 (version 2020.2), which denoises reads with single-nucleotide resolution based on error modeling. Chimeric sequences were detected and removed during this process. For downstream diversity analysis, rarefaction was conducted based on the minimum sequencing depth of 16,344 reads per sample, ensuring uniform depth across all samples. Taxonomic classification was performed using the Ribosomal Database Project (RDP) Classifier (version 2.13), implemented via QIIME2’s classify-consensus-vsearch method, with a minimum confidence threshold of 0.8. The NT_16S database (version 20221012) was used as the reference database.

Microbiota-related data analyses were performed using the Majorbio Cloud Platform.^1^ The Shannon index at the species level was calculated using Mothur software. The similarity among the microbial communities was determined by principal coordinate analysis (PCoA) based on Bray–Curtis dissimilarity using R software. The Adonis test was used to assess statistical significance.

### 2.7 Gut metabolomic analysis

Gut content (30 ± 5 mg) was added to a 1.5 mL centrifuge tube with 240 μL solution (methanol:water = 1:1 (v:v)) containing 0.02 mg/mL internal standard (L-2-chlorophenylalanine) to extract metabolites. The samples were ground for 6 min (−10°C, 50 Hz) and sonicated at a low temperature for 30 min (5°C, 40 KHz). The samples were placed at −20°C for 30 min to precipitate the proteins. The samples were then centrifuged for 15 min (4°C, 13000 × *g*) and the supernatant was transferred to a sample vial for liquid chromatography–tandem mass spectrometry (LC–MS/MS) analysis. A pooled quality control sample was prepared by mixing 20 μL of supernatant from all samples.

LC–MS/MS analysis of samples was performed using a Thermo ultra-high-performance liquid chromatography (UHPLC)-Q Exactive HF-X system. The mobile phase comprised 0.1% formic acid in water:acetonitrile (95:5, v/v) (solvent A) and 0.1% formic acid in acetonitrile:isopropanol:water (47.5:47.5, v/v) (solvent B). The flow rate was 0.40 mL/min and the column temperature was 40°C. The injection volume was 3 μL. The optimal conditions were set as follows: source temperature, 425°C; sheath gas flow rate, 50 arb; Aux gas flow rate, 13 arb; ion-spray voltage floating (ISVF), −3,500 V in negative mode and 3,500 V in positive mode; and normalized collision energy, 20–40-60 eV rolling for MS/MS. The full MS resolution was 60,000, and the MS/MS resolution was 7,500. Data were acquired using the data-dependent acquisition (DDA) mode. Detection was performed over a mass range of 70–1,050 m/z.

The raw UHPLC–MS data were converted into a common format using Progenesis QI v3.0 software (Waters, Milford, MA, United States) through baseline filtering, peak identification, peak integration, retention time correction, and peak alignment. The data matrix containing the sample names, m/z, retention times, and peak intensities was then exported for further analysis. The metabolites were simultaneously identified by searching the database.

The metabolomic data were uploaded to the Majorbio Cloud Platform for preprocessing and data analysis. Metabolites with variable importance in the projection (VIP) > 1 and *p* < 0.05 were determined as significantly different metabolites obtained by the orthogonal partial least squares discriminant analysis model and Student’s *t-*test. Differential metabolites between the two groups were mapped to their biochemical pathways through metabolic enrichment and pathway analysis based on the Kyoto Encyclopedia of Genes and Genomes (KEGG) database.^2^ This process was implemented through the SciPy (Python).

## 3 Results

### 3.1 *Phocaeicola coprocola* benefits learning and memory behaviors in honeybees

To elucidate the effect of *P. coprocola* on brain-related behaviors, honeybees were subjected to a standardized gut microbiota, followed by a 3-day exposure to *P. coprocola* or blank control, and an additional 2-day washout period. Learning and memory assays were performed on day 7 (Figures 1A,B). The *P. coprocola* group showed a better learning efficiency and ability. Following five rounds of training, the correct response rate was significantly higher in the *P. coprocola* group (43/58, 74.13%) than in the control group (30/59, 50.85%) (*p* = 0.0093, two-sided Chi-square test; Figure 1C). In the memory test, the proportion of honeybees that accurately identified and responded to odor stimuli was higher in the *P. coprocola* group (34/58, 58.62%) than in the control group (24/59, 40.67%), although this difference was not statistically significant (*p* = 0.0523, two-sided Chi-square test; Figure 1D).

### 3.2 *Phocaeicola coprocola* altered the intestinal flora of honeybees

**Figure 2:**
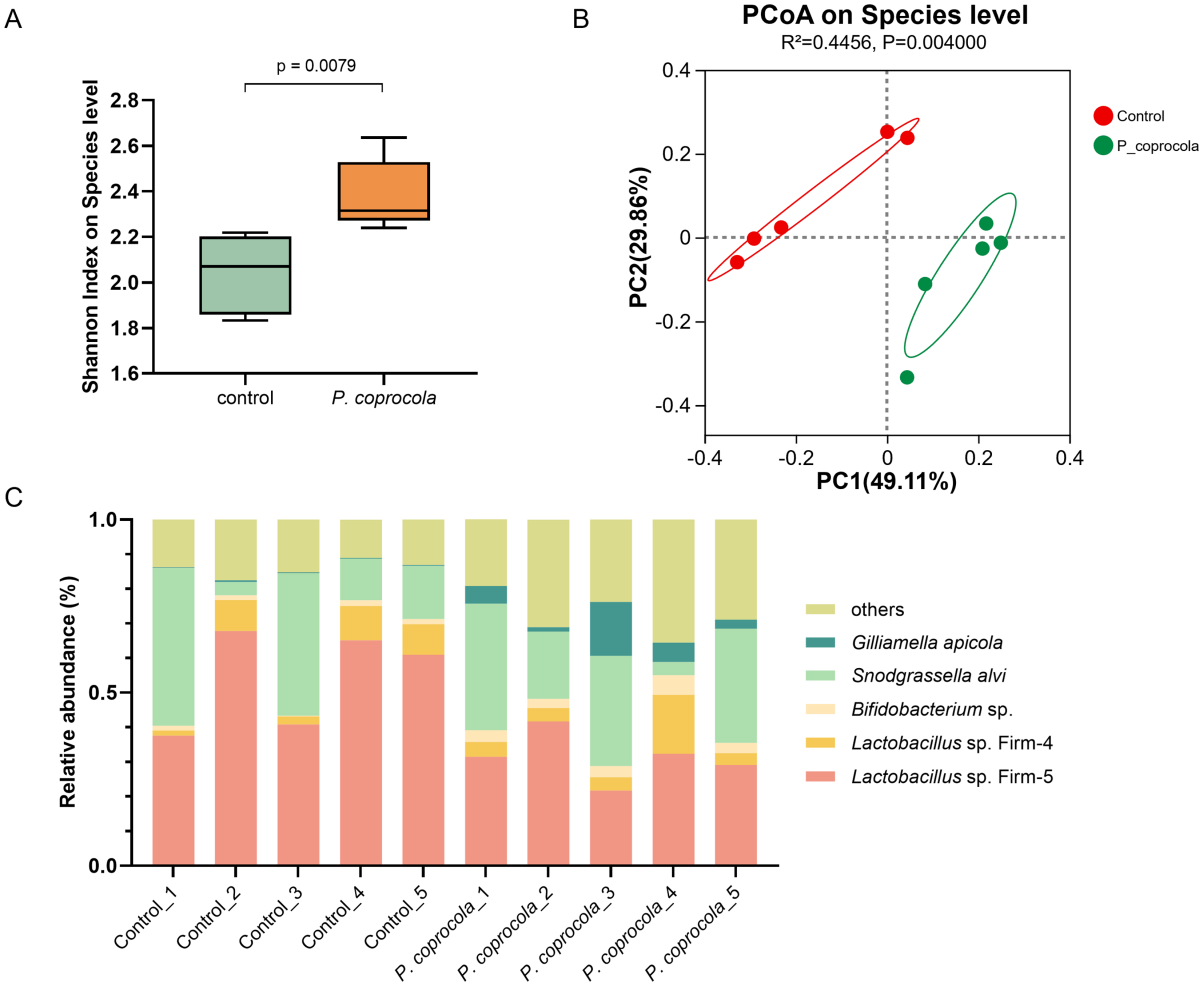
*Phocaeicola coprocola* alters the intestinal microbiota of honeybees. **(A)** Shannon diversity index at the species level in the honeybee gut microbiota showed a significant difference between the control and *P. coprocola* groups. A two-sided Mann–Whitney U test was used for statistical analysis (*p*-value shown in the panel). **(B)** PCoA based on Bray–Curtis dissimilarity demonstrates clear separation in gut community composition between the two groups. PERMANOVA (Adonis test) was applied to assess statistical significance (*R^2^* and *p*-value reported). **(C)** Relative abundance of core gut members in honeybees in the different samples.

The honeybee gut microbiota was analyzed using 16S rRNA gene sequencing. Notably, although the human-derived gut microbe *P. coprocola* did not successfully colonize the honeybee gut, the temporary transition had a profound effect on the microbial community. The alpha diversity of the *P. coprocola* group was higher than that of the control group, suggesting that *P. coprocola* can increase the species richness and evenness of the honeybee gut microbiota to some extent (Figure 2A). Beta diversity analysis revealed significant differences in the gut microbial community composition between the two groups (Figure 2B). *P. coprocola* altered the abundance and composition of the core gut microbiota in honeybees (Figure 2C). Specifically, the relative abundances of *Lactobacillus* Firm-5 and Firm-4 were reduced in the *P. coprocola* group, whereas those of *Bifidobacterium* sp. and *G. apicola* were increased. In addition, the proportion of species other than the core gut members increased significantly in the *P. coprocola* group.

### 3.3 *Phocaeicola coprocola* affected intestinal metabolites in honeybees

**Figure 3:**
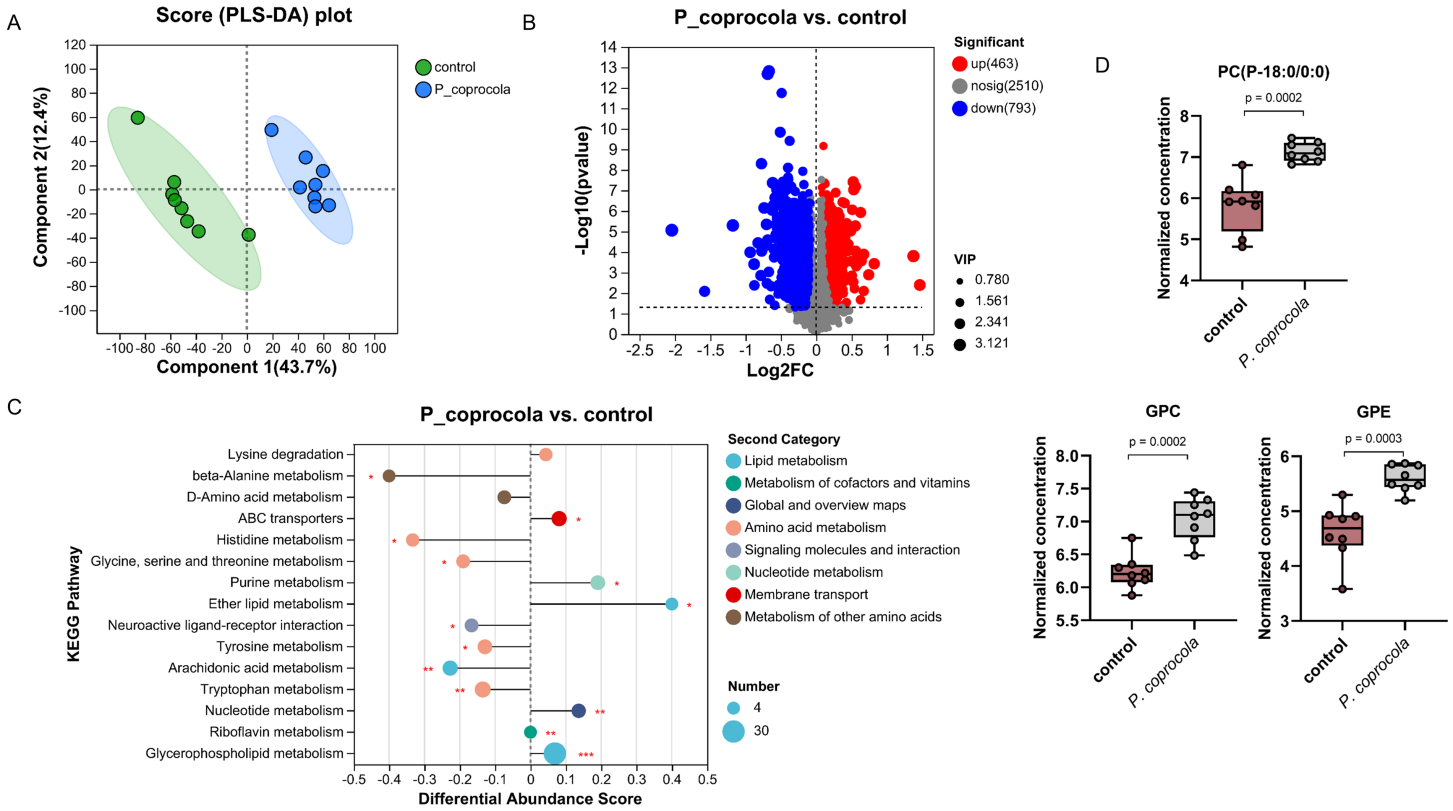
*Phocaeicola coprocola* affects intestinal metabolite profiles in honeybees. **(A)** PLS-DA based on all metabolites detected in the gut of honeybees. The abscissa is the score of the sample on the first principal component, and the ordinate is the score of the sample on the second principal component. **(B)** Volcano plot showing the differentially regulated metabolites between the two groups. The abscissa is the fold-change value of the difference in metabolite expression between the two groups, and the ordinate is the statistical test value of the difference. Each dot represents a metabolite and its size indicates the VIP value. Red dots indicate significantly upregulated metabolites, blue dots indicate significantly downregulated metabolites, and gray dots indicate nonsignificant differences. **(C)** DA scores of KEGG pathways, which reflect the overall change in all metabolites of the pathway. The length of the line segment indicates the absolute value of the DA score. The size of dots indicates the number of differentially expressed metabolites annotated in the pathway. Dots distributed on the right side of the central axis and the longer line segment indicate that the overall expression of the pathway tended to be upregulated and vice versa. *p < 0.05, **p < 0.01, ***p < 0.001. **(D)** Typical differential lipid metabolites in the guts of honeybees in the control and *P. coprocola* groups. Differences between groups were determined using the two-sided Mann–Whitney U test (*p* values shown in the panel).

Non-targeted metabolomics was used to analyze the intestinal contents of honeybees. Partial least squares discriminant analysis revealed a distinct separation of intestinal metabolites in honeybees between the two groups (Figure 3A). Compared to the control group, 1,256 metabolites from the *P. coprocola* group were identified, including 463 upregulated and 793 downregulated metabolites (Figure 3B). The differential abundance (DA) score of KEGG pathway enrichment was used to compare the pathways between the two groups (Figure 3C). Compared to the control group, the upregulated pathways in the *P. coprocola* group focused on lipid metabolism, such as glycerophospholipid and ether lipid metabolism, whereas downregulated pathways focused on amino acid metabolism, such as tryptophan and tyrosine metabolism.

Lipids are crucial components of cellular functions. They play an important role in maintaining membrane structure and regulating the function of nerve cells. Therefore, a two-sided Mann–Whitney U test was used to determine the differential metabolites between the groups in terms of the substances in the relevant pathway (Figure 3D; Supplementary materials). A total of 32 differential metabolites were enriched in glycerophospholipid and ether lipid metabolism, with DA scores > 0, indicating an overall upregulation of these pathways in the *P. coprocola* group compared to the control group. Phosphatidylcholine (PC)(14:0/16:1) and its metabolites lysoPC(P-18:0/0:0), PC(15:0/0:0), lysoPC(18:3), lysoPC(16:1/0:0), lysoPC(16:0/0:0), lysoPC(P-18:0/0:0), lysoPC(17:0/0:0), lysoPC(15:0/0:0), lysoPC(20:4/0:0), and glycerophosphocholine (GPC) were significantly upregulated in the *P. coprocola* group. Two phosphatidylethanolamines (PE) were detected in the *P. coprocola* group: PE(16:1/P-18:1) was downregulated and PE(16:1/14:1) was upregulated. The PE derivatives phosphatidyl-N-methylethanolamine (PE-NMe 24:0/22:5, PE-NMe2 18:4/16:0, PE-NMe2 22:1/22:4, and PE-NMe2 20:5/14:1) were downregulated, whereas glycerophosphoethanolamine (GPE) was significantly upregulated in the *P. coprocola* group. Phosphatidate (PA) changes were not consistent between the two groups, with 10 PAs upregulated in four (PA(20:3/15:0), PA(i-12:0/i-14:0), PA(8:0/12:0), and PA(10:0/i-15:0)) and downregulated in six (PA(18:1/20:5), PA(10:0/i-16:0), PA(10:0/a-17:0), PA(20:2/20:0), PA(18:2/16:0), and PA(18:3/16:0)) of the *P. coprocola* groups.

### 3.4 *Phocaeicola coprocola* affected lipid metabolism by altering the intestinal flora of honeybees

**Figure 4:**
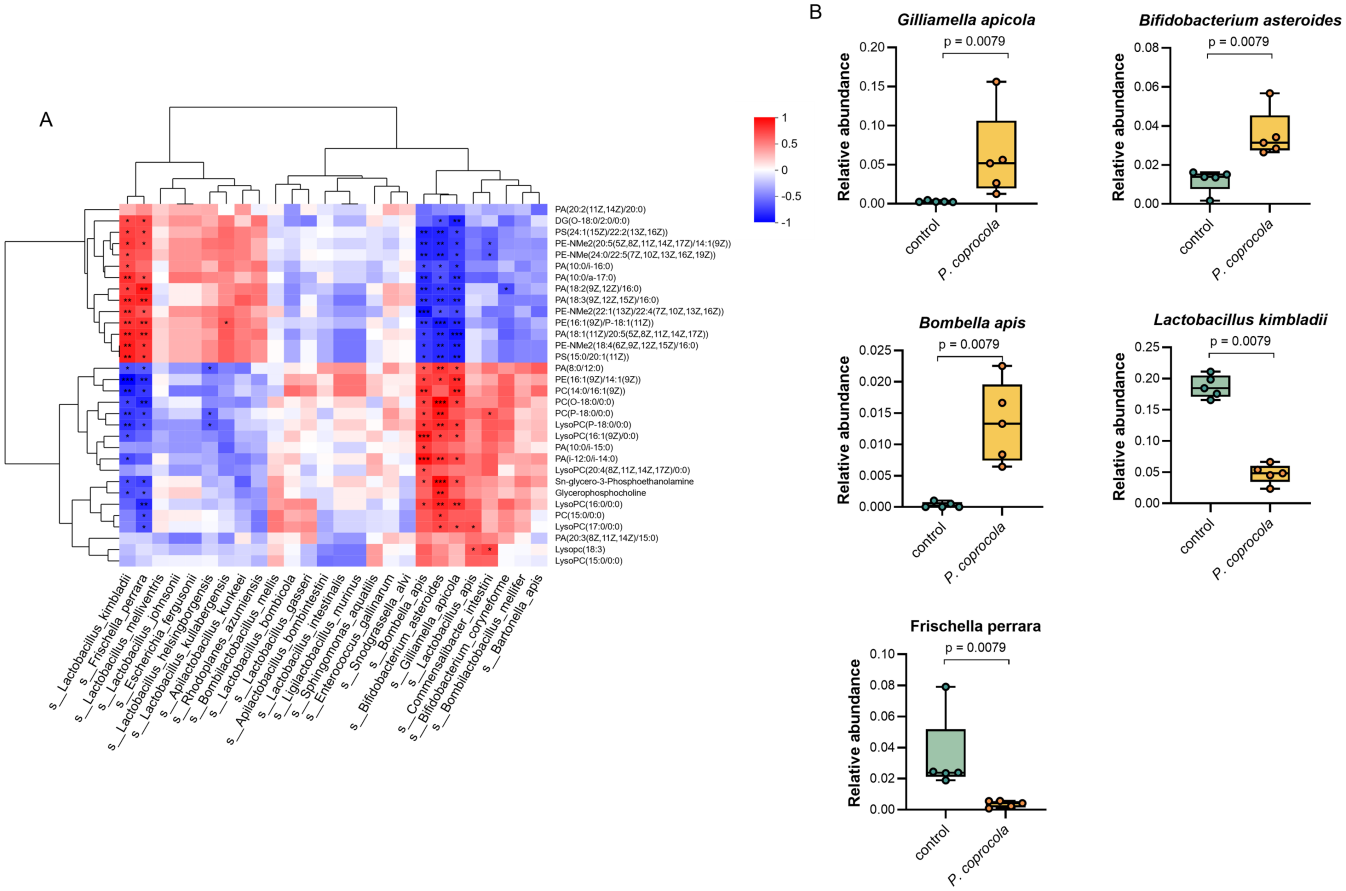
*Phocaeicola coprocola* influences lipid metabolism by modulating the gut microbiota of honeybees. **(A)** Heat map of Spearman’s correlation coefficients between differential lipid metabolites and microbial species in the guts of honeybees. Red and blue colors represent positive and negative correlations, respectively. Color intensity is proportional to Spearman’s rank correlation values. *p < 0.05, **p < 0.01, ***p < 0.001. **(B)** The relative abundance of bacterial species significantly correlates with differential lipid metabolites in the control and *P. coprocola* groups. Differences between groups were determined using the two-sided Mann–Whitney U test (*p*-values shown in panel).

To further explore the potential mechanisms and identify the key microbiota and metabolites through which *P. coprocola* enhances learning and memory in honeybees, a heat map of Spearman’s correlation coefficients between the differentially expressed lipid metabolites and microbial species was plotted (Figure 4A). The relative abundances of *G. apicola*, *Bifidobacterium asteroides*, *Bombella apis*, *Lactobacillus kimbladii*, and *Frischella perrara* showed a clear positive or negative correlation with differential lipid metabolites (Figure 4B). These bacterial species showed varying shifts in abundance following *P. coprocola* supplementation. *G. apicola*, *B. asteroides*, and *B. apis* increased and positively correlated with PCs, lysoPCs, GPC, and GPE; *L. kimbladii* and *F. perrara* decreased and negatively correlated with the above metabolites. These results suggest that *P. coprocola* may affect lipid metabolism by altering the abundance of *G. apicola* and *B. apis* in the gut of honeybees, thereby enhancing learning and memory.

## 4 Discussion

Evidence indicates that the trillions of intestinal microorganisms help regulate the gut-brain axis, and neuropsychiatric disorders are closely linked to the gut microbiome (Rutsch et al., 2020; Socała et al., 2021). Therefore, the potential impact of probiotics on brain function should not be overlooked (Sorboni et al., 2022). In this study, we used honeybees, a simple preclinical model, to investigate the effects and underlying mechanisms of action of *P. coprocola* on brain-related behaviors.

Our results showed that supplementation with *P. coprocola* improved the learning and memory abilities of honeybees. Interestingly, this bacterium did not appear to colonize the honeybee intestine. This outcome aligns with the concept of host specificity, which describes the restriction of certain microorganisms to particular host species and has been widely observed across diverse animal gut microbiomes. Honeybees, in particular, exhibit a highly selective gut environment that favors a conserved set of core symbionts, often showing specificity down to the strain level (Guo et al., 2023). Recent studies have emphasized that this specificity arises from multiple factors, including host immune filtering, physiological compatibility, and priority effects, which together maintain a stable and host-adapted microbial community in social bees (Mazel et al., 2025). The inability of a human-derived strain like *P. coprocola* to establish colonization in the honeybee gut is thus consistent with these findings. Nevertheless, repeated administration of high doses of *P. coprocola* had a pronounced and lasting impact on the honeybee gut microbiome. Despite its transient presence, *P. coprocola* appears to have reshaped the microbial community by temporarily disrupting the original equilibrium. This bacterium is equipped with sophisticated polysaccharide utilization loci that allow it to degrade complex plant-derived carbohydrates, such as cellulose (Zafar and Saier, 2021). The breakdown products are not only used by *P. coprocola* itself but also become accessible to other gut microbes, acting as a metabolic subsidy. This process may promote the expansion of key native taxa such as *Gilliamella apicola* and *Bifidobacterium asteroides*, initiating a new, more diverse microbial configuration through cross-feeding and niche modification (Kwong et al., 2014). Moreover, strain-level analyses in honeybee gut symbionts have shown that local adaptation significantly enhances colonization efficiency and metabolic performance within specific hosts, and that exposure to non-native strains can still elicit host transcriptional responses (Zhou et al., 2025). Thus, even a non-colonizing species like *P. coprocola* could transiently modulate host immunity or gut physiology in ways that support longer-term ecological restructuring of the microbiome.

Small-molecule metabolites are important mediators of gut-brain communication. Metabolomic analysis of the intestinal contents revealed that *P. coprocola* significantly altered lipid metabolism in honeybees, especially in the glycerophospholipid metabolic pathways. Lipids play a crucial role in brain function, and their vital importance in tissue physiology and cellular signaling has been well documented in studies on neurological disorders (Yoon et al., 2022). Glycerophospholipid supplementation enhances cognition and supports brain structure in humans, rats, and mice (Reddan et al., 2018). Specific gut microbial mono-colonization in bumblebees can induce an increase in glycerophospholipids in the gut and subsequent accumulation in the hemolymph; these metabolites are naturally transported via the open circulatory system to the brain, resulting in better memory performance (Li et al., 2021).

In the present study, significant increases in PCs, lysoPCs, and GPC were observed in the *P. coprocola* group. PCs and their derivatives play critical roles in biofilm formation and synaptic functions. An aging cohort of 560 elderly individuals showed that plasma PC and lysoPC levels were significantly associated with cognitive and motor functions (Tian et al., 2024). In addition, the dietary intake of exogenous PC has positive effects on cognition. Ylilauri et al. (2019) conducted a prospective study with nearly 2,500 participants, and the results showed that higher PC intake was associated with better performance in verbal fluency and memory functions. GPC is a precursor of acetylcholine, which is an important neurotransmitter in the brain. Levels of GPC are lower in the plasma of healthy elderly individuals than in healthy young individuals and lower in patients with dementia than in healthy elderly individuals, indicating a positive impact of GPC on cognition (Teruya et al., 2021). In some countries, GPC has been approved as a drug or nutraceutical agent for cognitive improvement in patients with craniocerebral injuries and dementia. A meta-analysis that included seven randomized controlled trials and one prospective cohort study concluded that the use of *α*-GPC alone or in combination with ChE-I donepezil improves the cognition, functional, and behavioral status of patients with Alzheimer’s disease and other dementias of neurological origin (Sagaro et al., 2023). In addition to PCs, we detected significant, although inconsistent, changes in the two PEs between the two groups, and the related metabolite GPE was significantly upregulated in the gut of the *P. coprocola* group. *In vitro* studies have demonstrated that GPE exerts neuroprotective effects on human hippocampal neurons by increasing acetylcholine levels, attenuating lipid peroxidation, and enhancing autophagy (Daniele et al., 2020). In addition, the results of hypothalamic lipid profiling in mice suggest that GPE may serve as an important marker for determining cognitive impairment (Wackerlig et al., 2020). Our results indicate that *P. coprocola* affects lipid metabolism in the honeybee gut by increasing the glycerophospholipid pools. These lipids may directly or indirectly affect brain function through the circulatory system, thereby enhancing learning and memory in honeybees.

How does *P. coprocola* affect the intestinal lipid metabolism? Correlation analyses of the flora and metabolites provided partial clues. Five bacterial species, *G. apicola*, *B. asteroides*, *B. apis*, *L. kimbladii*, and *F. perrara*, were significantly correlated with glycerophospholipid levels. The first three exhibited positive associations with these neuroprotective lipids and showed marked increases after supplementation with *P. coprocola*, whereas the last two displayed negative correlations and notable decreases in the *P. coprocola* group. A high consistency in bacterial and metabolite changes was observed. A study on gnotobiotic honeybees confirmed a significant correlation between *G. apicola* and hemolymph glycerophospholipid metabolism (Zhang et al., 2022). Moreover, *G. apicola* cross-feeds with *S. alvi* (Kwong et al., 2014), which digests polysaccharides synergistically with *Bifidobacterium* spp. (Zheng et al., 2019) and tends to form a symbiotic network. The abundance of these bacteria increased simultaneously in the *P. coprocola* group. Our results suggest that the altered lipid metabolic profiles are, at least in part, a result of the effects of *P. coprocola* supplementation on the structure and function of honeybee gut microbes.

Overall, the present study introduces a novel preclinical platform and demonstrates that supplementation with *Phocaeicola coprocola* improves cognitive performance in honeybees. This beneficial effect appears to stem from its modulation of the gut microbiota, which in turn influences the composition and function of the microbial community. Notably, *P. coprocola* supplementation led to increased levels of lipid molecules such as PCs, GPC, and GPE, which are involved in maintaining neuronal membrane homeostasis and regulating intracellular signaling cascades.

These findings highlight the significant potential of *P. coprocola* as a probiotic candidate and suggest a promising avenue for enhancing cognitive function through microbiota–gut–brain interactions. However, several limitations should be acknowledged. First, although *P. coprocola* supplementation was associated with improved learning and memory in honeybees, the current findings are correlational. Specifically, the observed shifts in gut lipid metabolism may be linked to behavioral outcomes, but no direct causal mechanisms have been established. Future studies employing functional assays, such as neurotransmitter profiling, targeted lipid manipulation, or brain-level analyses, will be necessary to determine whether specific gut-derived lipids mediate these cognitive effects. Second, while honeybees offer a genetically tractable and cost-effective model with conserved brain-related pathways, their evolutionary distance from humans limits the direct translational relevance. Further validation in mammalian systems will be critical. Finally, the inability of *P. coprocola* to establish stable colonization in the honeybee gut highlights the evolutionary and ecological constraints imposed by host-specific compatibility. Nevertheless, our findings demonstrate that even transient microbial exposures can induce durable shifts in the gut ecosystem. These results emphasize the need to account for both colonization potential and host specificity when interpreting microbiota-host interactions, particularly in cross-species or probiotic research contexts.

## Data Availability

The 16S rRNA gene sequencing data have been deposited into NCBI Sequence Read Archive (SRA) under BioProject PRJNA1219696. Sequencing reads are publicly accessible, with accession numbers detailed in the Supplementary materials. Metabolomics data have been deposited into CNGB Sequence Archive (CNSA) of China National GeneBank DataBase (CNGBdb) with accession number CNP0006879. All data supporting the findings of this study are available in the manuscript or Supplementary materials.

## Footnotes

^1^ https://cloud.majorbio.com

^2^ http://www.Genome.jp/kegg/
